# Q_AI/ML-SaMD_: A Hybrid Health–Technology Quantifiable Quality Metric for Artificial Intelligence/Machine Learning-Based Software as a Medical Device

**DOI:** 10.3390/healthcare14162587

**Published:** 2026-08-17

**Authors:** Shouki A. Ebad

**Affiliations:** Center for Scientific Research and Entrepreneurship, Northern Border University, Arar 73213, Saudi Arabia; shouki.abbad@nbu.edu.sa

**Keywords:** AI/ML, SaMD, AI/ML-SaMD, quality, measurement

## Abstract

**Background:** The increasing integration of Artificial Intelligence (AI) and Machine Learning (ML) into medical devices necessitates robust quality evaluation methods. However, existing approaches remain qualitative, sector-specific, or focused on isolated attributes, leaving a gap in quantifiable assessment for AI/ML-driven Software as a Medical Device (SaMD). **Objective**: This study introduces Q_AI/ML-SaMD_, a novel hybrid metric that provides a comprehensive, quantifiable measure of AI/ML-SaMD quality by synthesizing health and information technology (IT) dimensions into a single composite, benchmark-ready score. **Methods**: The metric integrates key attributes from a systematic literature review, classified into Health and IT domains. Sub-metrics (Q_Health_ and Q_IT_) use weighted sums, while the overall score employs a Weighted Geometric Mean with configurable parameters to penalize domain imbalances. Validation included (a) theoretical validation against four mathematical properties, (b) an illustrative example with sensitivity analysis, (c) expert-based validation with six specialists, and (d) an evidence-based case study on FDA-authorized IDx-DR using public regulatory and clinical documentation. **Results**: The illustrative example yielded a score of 29.7 (“Unsuitable”). Sensitivity analysis confirmed robustness across weight, score, and combined uncertainty perturbations, with classification unchanged. Expert validation showed 83.3% agreement. The IDx-DR case study produced a score of 82.3 (“Admissible”), correctly aligning with the device’s regulatory status and supporting external validity. **Conclusions**: The Q_AI/ML-SaMD_ metric provides a foundational, quantifiable framework for AI/ML-SaMD quality assessment, bridging qualitative regulatory principles and measurable outcomes. It offers a practical tool for developers, regulators, and clinicians to benchmark and track quality across the SaMD lifecycle.

## 1. Introduction

Software as a Medical Device (SaMD) is medical-purpose software that works on its own without needing a physical device [1]. It runs on common platforms like smartphones or computers, not requiring specialized hardware. Artificial Intelligence (AI) involves programming machines to mimic human cognitive abilities, such as reasoning, problem-solving, and learning [2]. Machine Learning (ML) is a field of AI where computers learn from data without being explicitly programmed [2]. AI/ML-based software as a medical device (AI/ML-SaMD) refers to standalone software utilizing ML and other AI techniques to perform medical functions without being part of a physical device [3]. Examples include tools for image analysis in radiology, predictive analytics for chronic disease management, and decision support systems for clinicians. In fact, the number of approved AI/ML-SaMDs has increased over the last few years [4]. This interest stems from the fact that applying AI/ML techniques to software-intensive systems produced promising and encouraging results [2].

However, the deployment of AI/ML-SaMD in real-world healthcare settings necessitates stringent quality assurance to ensure they perform reliably and ethically. From a software perspective, quality refers to the overall attributes of a software product that determine how well it meets the requirements identified by its users [5]. To the best of our knowledge, there is a lack of measurement in this field to report the quality and strength of AI/ML-SaMDs, and it remains unclear how high-quality these medical devices are [6]. Measuring such quality is not only useful but also necessary because the interested community cannot tell whether the device is effective if there is no measure of its attributes. Therefore, measurement is required for at least evaluating and controlling this type of device because, as DeMarco’s rule states, “you can neither predict nor control what you cannot measure’’ [7].

Although measurement is nothing more than a number or symbol that is assigned to the medical device to characterize its quality [8], the quality of AI/ML-SaMD stems not only from technological advancements but also from other sources such as healthcare providers and governmental agencies. Therefore, research in this domain spans various fields, including social care, welfare, bioengineering, AI, machine learning, software development, and citizen science [9]. From the literature, the most common quality attributes come from two sources: IT and health [3]. While AI/ML-SaMD is becoming more common, a key challenge remains—there is an absence of a quantifiable, holistic score that combines technical and clinical quality metrics into one comparable score. Current methods are typically qualitative (consensus-based frameworks), specific to a particular sector (radiology), or narrow to specific characteristics (transparency or performance) [6]. This divide hinders accurate measuring, decision-making and quality-oriented growth. To bridge this gap, this paper presents a novel hybrid metric—called Q_AI/ML-SaMD_—that combines key factors from both health and information technology (IT) and provides a composite score ready for benchmarking. This paper directly addresses that gap and extends the prior work by making the following key contributions:

- Proposing a novel, structured metric that moves beyond a simple list of attributes to a quantifiable scoring framework, integrating both health-specific and IT-specific factors
- Providing a quantifiable scoring metric, including factor classification, weight assignment, and score aggregation.
- Empirically validating the metric through a multi-faceted analysis: (a) demonstrating its calculation and sensitivity via a controlled illustrative example, (b) providing expert-based validation to establish face validity and practical relevance, and (c) conducting an evidence-based case study by applying the metric to an FDA-authorized AI/ML-SaMD (IDx-DR), demonstrating its ability to produce a score that correctly classifies a real-world system and aligns with its regulatory status.

The rest of this paper is organized as follows: Section 2 presents a review of related works. Section 3 introduces our new universal metric for quantifying AI/ML-SaMD quality. The validation of the proposed metric against theoretical properties is discussed in Section 4. Section 5 presents empirical validation, comprising an illustrative example, a sensitivity analysis, an expert review, and an evidence-based case study applying the metric to an FDA-authorized AI/ML-SaMD product. In Section 6, we discuss some threats to the study’s validity. Finally, the last section presents concluding remarks and offers some directions for future work.

## 2. Related Work

The regulatory and quality assessment landscape for AI/ML-SaMD is rapidly evolving, with recent research coalescing around three key themes: foundational standards, persistent challenges, and the pursuit of harmonized solutions.

### 2.1. Foundational Standards and Regulatory Frameworks

A critical foundation lies in understanding how risk management integrates into the regulatory process. Odaibo (2021) [10] establishes this connection by demonstrating how ISO 14971 [11] underpins broader regulatory frameworks for AI/ML-SaMD. This work is pivotal as it grounds the often-abstract discussion of AI ethics in an established, practical standard for medical device safety. Complementing this, reviews by Giansanti (2022) [12] provide a geographical breadth, analyzing international approaches and China’s specific regulatory trajectory, respectively. These studies consistently highlight a focus on ethical issues, algorithm transparency, and the challenges of managing post-market changes, underscoring a global struggle to adapt traditional regulatory models to adaptive AI systems. Five attributes (accuracy, AI model transparency, safety, performance, and specificity and sensitivity) are the most frequently used criteria for assessing the quality of AI-SaMD, according to our previous systematic analysis [3]. This analysis also highlighted a significant gap in the current research landscape, particularly the absence of a universal metric to comprehensively evaluate the quality of AI-SaMD.

### 2.2. Identification of Persistent Challenges

Beyond frameworks, a significant body of literature is dedicated to diagnosing the ongoing hurdles in AI/ML-SaMD regulation. Chothani et al. (2022) [6] succinctly catalogues enduring issues such as cybersecurity, decommissioning protocols, and the high costs of complex development. This list of challenges is given clinical context by Hwang et al. (2021) [13], who, through the lens of chest radiography, illustrate the practical difficulties of implementing these tools in real-world clinical practice. A common thread, as identified by Reddy (2024) [14], is the lack of consistent global regulations, which creates a fragmented market and complicates international collaboration. The urgency of this problem is underscored by Yang et al. (2024) [15], who note the growing number of FDA-cleared AI/ML-SaMD products, thereby increasing the immediate need for robust and scalable oversight mechanisms.

### 2.3. Moving Towards Solutions: Frameworks and Consensus

In response to these challenges, researchers have begun proposing concrete methodological solutions. A notable contribution is the “Simulation for Regulation of SaMD” (SRS) framework by O’Driscoll et al. (2024) [16]. Developed via international expert consensus, the SRS framework provides a structured set of criteria for evaluating clinical simulations, directly addressing concerns like simulation fidelity and bias. This represents a significant step towards standardizing pre-market validation. Similarly, Reddy (2024) [14] call for international standards, global data security protocols, and enhanced post-market surveillance, moving the discourse from identifying problems to proposing systemic fixes. Klavetter et al. [17] posit that the implementation of AI/ML-SaMD for disease detection has the potential to be as transformative as key historical medical breakthroughs: the establishment of epidemiology (1831), the discovery of penicillin (1928), the elucidation of the double-helix structure (1953), and the emergence of gene therapy (1990s). These preceding innovations fundamentally reshaped patient care, health systems, and the market.

### 2.4. The Critical Gap in Quality Quantification

While the aforementioned research effectively maps the regulatory terrain and proposes high-level frameworks, a critical gap remains in the quantification of overall quality. Current work tends to be qualitative (e.g., consensus criteria [16]), sector-specific (e.g., radiology [13]), or focused on discrete issues like transparency or risk management. There is a lack of a universal, quantitative metric that can synthesize the multitude of health-related (e.g., clinical efficacy, safety) and IT-related (e.g., robustness, cybersecurity) factors into a single, comparable score. This missing tool makes it difficult for researchers, regulators, and developers to quantitatively assess, compare, and benchmark the holistic quality of different AI/ML-SaMD products. Mentzou et al. [18] explore existing evidence on AI-enabled digital self-diagnosis tools (symptom checkers) to identify common themes and establish an interdisciplinary research agenda. It reveals significant gaps in understanding their development, implementation, impact, and oversight, urging multidisciplinary efforts. The terminology used to describe these tools and their underlying technologies also varies widely. Our work aims to bridge this precise gap. We build upon the foundational challenges identified by Chothani et al. [6] and the call for standardization by [14,17], but we move beyond qualitative frameworks to provide a universal metric. Our previous systematic analysis [3] identified a significant research gap: the lack of a universal, comprehensive metric for AI-SaMD evaluation.

### 2.5. Positioning of the Proposed Q_AI/ML-SaMD_ Metric

While the existing frameworks provide essential qualitative guidance or focus on specific life cycle stages, the proposed Q_AI/ML-SaMD_ metric offers a distinct quantitative contribution. Unlike high-level regulatory principles such as International Medical Device Regulators Forum (IMDRF) guidelines or process-oriented standards (e.g., ISO 14971 for risk management [11]), Q_AI/ML-SaMD_ provides a composite, scorable output that synthesizes both health and IT factors into a single comparable value. In contrast to domain-specific evaluation frameworks such as the Simulation for Regulation of SaMD (SRS) [16], which offers criteria for validating clinical simulations, our metric is designed for holistic, summative assessment across the entire system, not solely its simulated performance. Furthermore, while previous work often treats technical and clinical attributes separately, Q_AI/ML-SaMD_ explicitly integrates them through a Weighted Geometric Mean (WGM) formula (Equation (1)), enabling stakeholders to quantify trade-offs and track overall quality improvement. This positions Q_AI/ML-SaMD_ not as a replacement for existing frameworks, but as a complementary quantitative tool that operationalizes their qualitative objectives into a measurable, benchmark-ready score. Drawing on established empirical software engineering traditions [19,20], our work seeks to provide a quantifiable, validated metric rather than a purely qualitative framework.

## 3. Measuring the AI/ML-SaMD Quality

Quantification of parameters that influence the overall quality is crucial for research in the field of software-intensive systems [21]. According to [3], there have been attempts to propose metrics to quantify a specific attribute in AI/ML-SaMD such as performance and accuracy. However, such metrics try to quantify a single quality attribute but not the whole, or the overall quality.

Combining multiple metrics into a composite metric is a recognized approach in systems engineering to provide a more comprehensive evaluation of quality. Therefore, a comprehensive evaluation of AI/ML-SaMD that involves a multi-faceted quality framework that combines multiple metrics could be suitable. To address quality considerations from an abstraction level, we propose a new quality metric of AI/ML-SaMD as the WGM of two functions: IT quality and health quality. The first function (IT quality) includes every technical aspect that has an impact on the AI/ML-SaMD. It might include factors such as AI/ML model, software, and connection. In contrast, the other function (health function) includes every health aspect (i.e., non-technical) that has an impact on the AI/ML-SaMD; it might include regulatory compliance, clinician familiarity, and user experience. The new metric of an AI/ML-SaMD, Q_AI/ML-SaMD_, is calculated as in Equation (1):

(1) $$Q_{\mathrm{AI/ML-SaMD}}\;=\;Q_{IT}^{\alpha }\times \;Q_{Health}^{\beta }$$

where α and β are relative importance weights for IT and Health, respectively; α + β = 1; default values: α = β = 0.5 (equal weighting). These can be calibrated based on stakeholder priorities for specific contexts. Particularly, α and β (default α = β = 0.5) represent the relative importance of the IT and Health dimensions. Equal default values are chosen to reflect the **balanced importance** of both domains in AI/ML-SaMD quality—neither technical robustness nor clinical/regulatory fitness should dominate a holistic assessment. However, these parameters are configurable: in context of prioritizing clinical safety (e.g., high-risk diagnostics), β may be increased; in context of focusing on technical innovation (e.g., early-stage prototypes), α may be increased. This flexibility ensures the metric’s adaptability without compromising its core structure.

To illustrate the effect of adjusting α and β, consider this baseline scenario (taken from Section 5.1), where Q_IT_ = 0.284 and Q_Health_ = 0.310, yielding Q_AI/ML-SaMD_ = 0.297 with α = β = 0.5. If the device is intended for a high-risk diagnostic application where clinical safety is paramount, a stakeholder might assign greater weight to the health dimension, e.g., α = 0.3, β = 0.7. The overall score then becomes Q_AI/ML-SaMD_ = 0.284^0.3^ × 0.310^0.7^ = 0.302 (scaled: 30.2). Conversely, for an early-stage prototype where technical performance is the primary focus, one might set α = 0.7, β = 0.3, yielding Q_AI/ML-SaMD_ = 0.284^0.7^ × 0.310^0.3^ = 0.292 (scaled: 29.2). In both cases, the classification remains “Unsuitable” (0–60), demonstrating that the metric is robust to reasonable shifts in domain weighting while still reflecting the intended prioritization. This numerical illustration confirms that the configurable parameters provide meaningful adaptability without undermining the metric’s consistency.

To estimate the Q_IT_ and Q_Health_ functions, we assume there are n and m factors contributing to the IT quality and health quality, respectively, with each factor having a weight w_i_ and a score s_i_. Accordingly, the Q_IT_ and Q_Health_ sub-metrics are calculated as in Equations (2) and (3), respectively:

(2) $$Q_{IT}=\sum_{i=1}^{n}w_{i}s_{i}$$

where s_i_ is the score for each IT factor on a scale of 0–1, and w_i_ is the relative importance weight assigned to each factor.

(3) $$Q_{Health}=\sum_{j=1}^{m}w_{j}s_{j}$$

where s_j_ is the score for each health factor, and w_j_ is the weight assigned to each factor.

The weights (wi) are defined as relative importance values that sum to **1** (or equivalently, **100** for percentage-based representation). When expressed as percentages (sum = 100), the same mathematical properties apply, and the Q scores are simply scaled by a factor of 100. This scaling does not affect relative comparisons or classification thresholds. In fact, the weighted sum formulation (Equations (2) and (3)) is chosen for its interpretability, additive nature, and alignment with standard composite metric practices in software engineering and health technology assessment [8,22]. Unlike multiplicative formulations, the weighted sum allows stakeholders to clearly identify which factors contribute most to quality deficits, enabling targeted improvement efforts. For example, suppose *Q*_IT_ = 0.95 and *Q*_Health_ = 0.10, where their weight is α = β = 0.5. Their simple average or weighted average is computed as (0.95 + 0.10)/2 = (0.95 × 0.5) + (0.10 × 0.5) = 0.525, whereas 0.95^0.5^ × 0.10^0.5^ = 0.308, which reflects an unbiased realistic integrated overall score. Furthermore, the sensitivity analysis in Section 5.2 demonstrates that the metric’s outcomes are robust to reasonable weight variations, mitigating concerns about the masking of low-scored factors.

### 3.1. On the Determination of Weights and Scores

For the overall quality score (Equation (1)), WGM is employed. Unlike the arithmetic mean, WGM penalizes imbalances between Q_IT_ and Q_Health_, ensuring that a critical failure in one domain cannot be completely masked by high performance in the other [23]. The parameters α and β represent the relative importance of the IT and Health dimensions, respectively, with equal default weights (α = β = 0.5) that can be adjusted to reflect context-specific priorities. The application of the proposed Q_AI/ML-SaMD_ metric follows a two-phase process to ensure both contextual relevance and evaluative consistency:

- Weight Calibration Phase: Before evaluation, the importance weights w_i_ for each factor must be established for the specific deployment context. These weights should reflect stakeholder priorities (e.g., clinical, regulatory, technical) and can be determined through structured consensus methods such as the Delphi technique or the Analytic Hierarchy Process (AHP). This ensures the weighting scheme is objective, transparent, and aligned with the intended use of the AI/ML-SaMD system.
- Assessment and Improvement Phase: Once calibrated, the weights are held constant to maintain a stable reference framework. The quality scores s_i_ are then assigned based on evidence of the system’s actual performance (e.g., validation results, audit reports, or compliance documentation). In this phase, improvements in Q_AI/ML-SaMD_ are modeled by increasing the performance scores s_i_ while keeping weights fixed, thereby simulating quality enhancement without altering the agreed-upon importance of the underlying factors.

This approach ensures that the metric is both adaptable to context (via initial stakeholder calibration) and consistent for evaluation and comparison (via fixed weights during assessment). Section 5.4 demonstrates this two-phase process in practice through an evidence-based evaluation of the IDx-DR system, where publicly available regulatory and clinical documents provide the basis for scoring.

### 3.2. Benchmarking

Table 1 presents the numeric quality interpretation thresholds provided by the Saudi Food and Drug Authority (SFDA) in their guidance on AI/ML-based medical devices [24]. The SFDA document specifies these thresholds as a medical device-dependent example, rather than a fixed regulatory rule, to assist in categorizing and benchmarking AI/ML-SaMD systems for evaluation and regulatory decision-making.

## 4. Theoretical Validation

To validate the overall quality metric (Q_AI/ML-SaMD_) theoretically, we will check whether it satisfies key mathematical properties [22]: non-negativity, normalization, null value, and maximum value. This approach follows established practices for validating software metrics, as demonstrated in prior work (e.g., [25]).

To check the four mathematical properties for Equation (1) and its components (Q_IT_ and Q_Health_), assuming that the scores s_i_ and s_j_ are within a defined range [0, Smax] (where S_max_ is 1 or 100), and the weights w_i_ and w_j_ are non-negative and sum to 1 within their respective summations (∑w_i_ = 1 and ∑w_j_ = 1).

1. **Non-Negativity:** a measure satisfies non-negativity if its value is always greater than or equal to zero.

For s_i_ and s_j_: The problem states scores are on a scale of 0–1 or 0–100, which means s_i_ ≥ 0 and s_j_ ≥ 0.
For w_i_ and w_j_: As “relative importance weights,” they are inherently non-negative (w_i_ ≥ 0, w_j_ ≥ 0).
For Q_IT_, since w_i_ ≥ 0 and s_i_ ≥ 0, their product (w_i_ × s_i_) is non-negative. The sum of non-negative terms is always non-negative. Thus, Q_IT_ ≥ 0.
For Q_Health_, similarly, w_j_ ≥ 0 and s_j_ ≥ 0, so Q_Health_ ≥ 0.
For Q_AI/ML-SaMD_, since Q_IT_ ≥ 0 and Q_Health_ ≥ 0, and α, β ≥ 0, the product $Q_{IT}^{\alpha }$ × $Q_{Health}^{\beta }$ is non-negative. Therefore, Q_AI/ML-SaMD_ ≥ 0.

2. **Null Value:** a measure should yield a value of zero when all its contributing factors are at their absolute minimum (indicating the lowest possible quality or absence of the measured attribute).

To avoid nullifying the entire metric in cases where a factor score is zero, a microscopic correction factor ε = 1 × 10^−9^ is applied:
  **s’ = max(s, ε), where ε = 1 × 10^−9^** (4)
This preserves mathematical stability while effectively representing negligible quality contributions. The minimum possible score for any factor s_i_ or s_j_ is a tiny value close to zero (ε). If all s_i_ = ε for Q_IT_:
  Q_IT_ = ∑(w_i_ × ε) = ε × ∑w_i_ = ε
If all s_j_ = ε for Q_Health_:
  Q_Health_ = ∑(w_j_ × ε) = ε × ∑w_j_ = ε
For Q_AI/ML-SaMD_, if Q_IT_ = ε and Q_Health_ = ε:
  Q_AI/ML-SaMD_ = ε^α^ × ε^β^ = ε^(α+β)^ = ε^1^ = ε
Thus, the metric approaches zero as scores approach zero.

3. **Max Value:** a measure should yield its maximum possible value when all its contributing factors are at their absolute maximum (indicating the highest possible quality).

Let S_max_ be the maximum possible score (either 1 or 100).
If all s_i_ = S_max_ for Q_IT_, assuming ∑w_i_ = 1 (as they are “relative importance weights”):
  Q_IT_ = ∑(w_i_ × S_max_) = S_max_ × ∑w_i_ = S_max_
If all s_j_ = S_max_ for Q_Health_, assuming ∑w_j_ = 1:
  Q_Health_ = ∑(w_j_ × S_max_) = S_max_ × ∑w_j_ = S_max_
For Q_AI/ML-SaMD_, if Q_IT_ = S_max_ and Q_Health_ = S_max_:
  Q_AI/ML-SaMD_ = (S_max_)^α^ × (S_max_)^β^ = (S_max_)^(α+β)^ = (S_max_)^1^ = S_max_

4. **Normalization:** a measure is normalized if its values consistently fall within a specified, bounded range, typically the same range as the individual input scores (e.g., [0, 1] or [0, 100]).

As established above, the minimum value for Q_IT_ is ε (close to 0) and the maximum is S_max_, so Q_IT_ ∈ [ε, S_max_] ≈ [0, Smax]. Similarly, Q_Health_ ∈ [0, S_max_].
For Q_AI/ML-SaMD_, the minimum possible value of Q_AI/ML-SaMD_ is when both Q_IT_ and Q_Health_ are at their minimum (ε):
  Q_AI/ML-SaMD_ = ε^α^ × ε^β^ = ε^(α+β)^ = ε ≈ 0
The maximum possible value of Q_AI/ML-SaMD_ is when both Q_IT_ and Q_Health_ are at their maximum (S_max_):
  Q_AI/ML-SaMD_ = (S_max_)^α^ × (S_max_)^β^ = S_max_^(α+β)^ = S_max_
Therefore, Q_AI/ML-SaMD_ ∈ [0, S_max_].

Accordingly, the Q_AI/ML-SaMD_ metric satisfies all four mathematical properties, confirming that the proposed metric is theoretically valid for estimating overall quality. The WGM formulation (Equation (1)) provides a robust foundation that penalizes imbalances between IT and Health quality dimensions, ensuring a fair assessment across different AI/ML-SaMD devices.

### Alignment with Regulatory and Quality Frameworks

This work is grounded in established software and measurement engineering principles. The Goal-Question-Metric (GQM) approach [26] provides a structured framework for defining measurable goals, which aligns with our process of selecting and categorizing quality attributes. Furthermore, the foundational work on software metrics validation by Kitchenham et al. [19] and the properties for evaluating software metrics by Weyuker [20] provide a rigorous theoretical basis for ensuring that metrics are meaningful, measurable, and interpretable.

The proposed Q_AI/ML-SaMD_ metric is designed to complement and operationalize existing regulatory standards, quality models, and ethical frameworks for AI/ML-SaMD. It does not seek to replace them, but rather to provide a quantitative, composite score that synthesizes their qualitative and process-oriented principles into a measurable outcome. This alignment ensures the metric is both interoperable with current regulatory practices and practically useful for stakeholders needing a holistic quality benchmark. As in Table 2, this structured alignment demonstrates that Q_AI/ML-SaMD_ is not an isolated construct but a bridging tool designed to quantify and consolidate objectives from diverse, established frameworks. This enhances its utility for developers aiming to meet multi-faceted requirements and for regulators seeking consistent, evidence-based summaries of device quality.

## 5. Empirical Validation

### 5.1. An Illustrative Example

It is important to note that the presented real-world example is illustrative in nature. Its primary purpose is to demonstrate the calculation mechanics and sensitivity of the Q_AI/ML-SaMD_ metric in a controlled, transparent manner. Therefore, we first present an illustrative example to demonstrate the metric’s calculation (this section). We then apply the metric in an evidence-based manner to a real-world, FDA-authorized AI/ML-SaMD, IDx-DR, to demonstrate its practical utility and external validity (Section 5.4).

**Figure 1:**
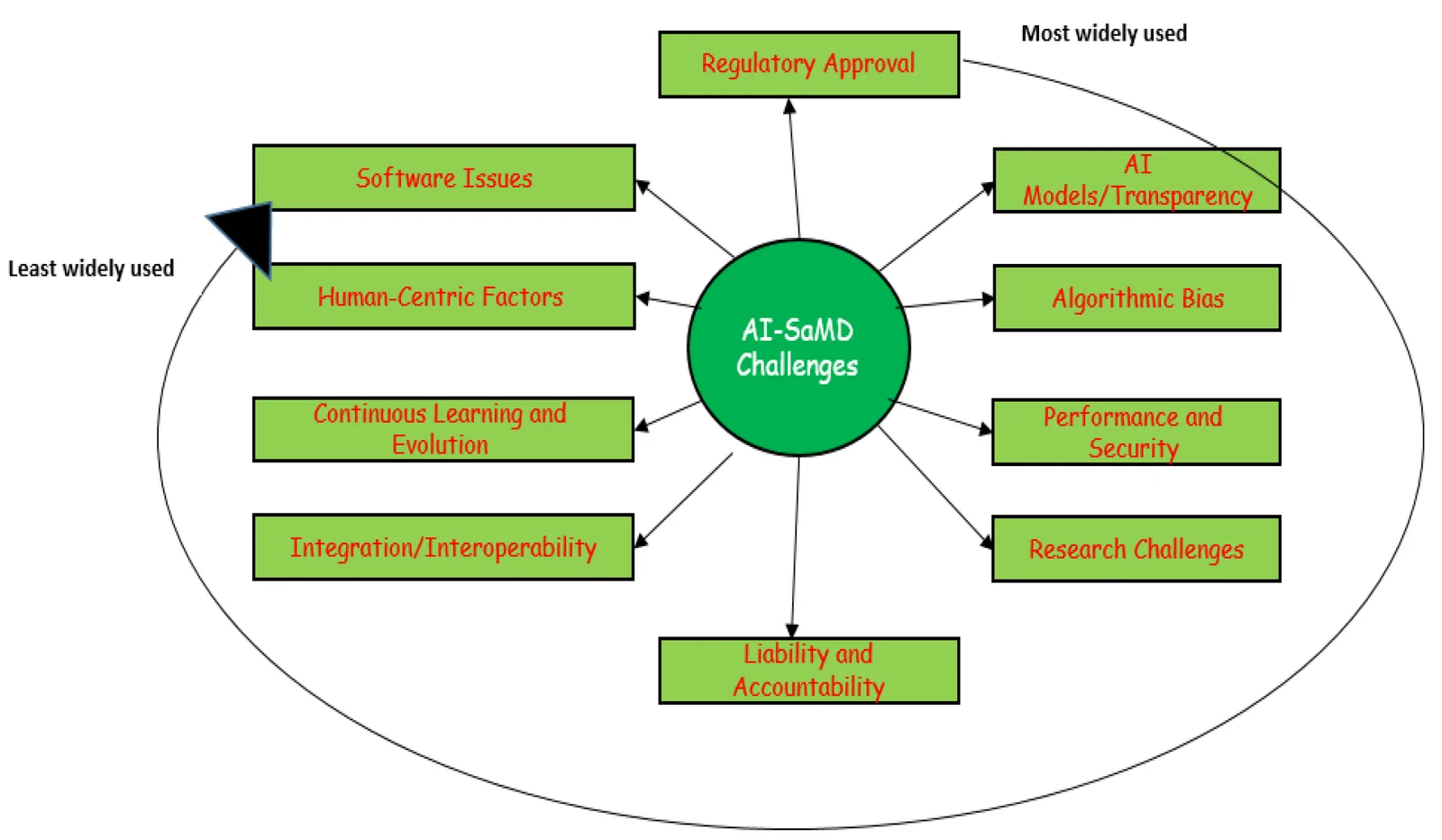
Top 10 quality attributes (or challenges) in AI/ML-SaMD implementation; adapted from [27].

The ten factors listed in Table 3 are derived from the key quality attributes (also referred to as challenges in the SaMD implementation context) identified in our prior systematic review [27] and illustrated in Figure 1. Throughout this paper, we use the terms ‘factors’, and ‘attributes’ interchangeably to refer to these ten quality dimensions. Regarding the assignment of weights, the importance weights w_i_ for each factor were derived from their relative ranking and prevalence as identified in the foundational literature [27]. The weights were then normalized so that the sum of weights for all factors within each type (IT and Health) equals 100 (equivalent to sum = 1 on a 0–1 scale). This scaling ensures that the contributions of individual factor scores s_i_ are proportionally scaled, maintaining a consistent and interpretable 0–100 scale for Q_IT_ and Q_Health_. To assess the robustness of the metric’s outcomes to these weight assignments, a sensitivity analysis is presented in Section 5.2. According to [27], the IT and healthcare communities have identified 10 key attributes that define a “good” AI/ML-SaMD, shown in Figure 1. These attributes, listed from most to least common, are regulatory approval, model transparency, algorithmic bias, performance and security, research challenges, liability and accountability, integration/interoperability, continuous learning and evolution, human-centric factor, and software issues.

To illustrate the weight derivation process, consider the frequency ranking from [27], where ‘Regulatory Approval’ appeared most frequently (ranked #1), followed by ‘Model Transparency’ (#2), and so on. Each factor’s weight was assigned proportionally to its relative prevalence in the literature, normalized to sum to 100. For health factors, ‘Regulatory Approval’ (rank #1) was assigned a high weight of 40, reflecting its dominance in the reviewed studies, while ‘Human-Centric Factors’ (rank #9) received a low weight of 10, reflecting lower emphasis. Similarly, for IT factors, ‘AI Models/Transparency’ (rank #2) was assigned a high weight of 22, reflecting its dominance in the present literature, while ‘Software Issues/Business’ (rank #10) received 10, reflecting lower emphasis. The exact mapping from rank to weight follows a linear descent, adjusted to maintain meaningful differentiation while ensuring the weights sum to 100. This transparent mapping ensures reproducibility and allows other researchers to adapt the weights based on updated literature or context-specific stakeholder input.

To apply the new metric (Equation (1)), we need to classify attributes into two distinct types: those primarily concerned with health and those more focused on IT. As in Table 3, the former class includes four quality attributes (regulatory approval, research challenges, liability and accountability, and human-centric factor), while the latter consists of six features (model transparency, algorithmic bias, performance and security, integration/interoperability, continuous learning and evolution, and software issues).

To find the Q_AI/ML-SaMD_ value, we assign weights based on importance (sum = 100 for each type). Next, we assign quality scores (0 to 1 scale). We then calculate the health and IT quality scores, Q_IT_ and Q_Health_. After that, we find the overall quality, Q_AI/ML-SaMD_ by taking the WGM of the Q_IT_ and Q_Health_ values. Table 4 provides details of these steps, using assumed scores and weights for demonstration.

As a result, the above result (Q_IT_ = 0.284, Q_Health_ = 0.310, and Q_AI/ML-SaMD_ = 0.297) indicates that the AI/ML-SaMD system is unsuitable because it shows low overall quality based on the evaluation criteria illustrated in Table 1.

In a high-quality scenario (i.e., increasing the value of Q_AI/ML-SaMD_), the scores (s_i_) are expected to be adjusted while keeping the weights (w_i_) fixed. This mechanism was chosen because weights represent the inherent importance of each factor, derived from challenge rankings, and altering them would misrepresent their significance. In contrast, scores reflect the actual performance of an AI/ML-SaMD system on each factor, so increasing them effectively simulates an improvement in quality without distorting the underlying importance structure. This method ensures that the quality simulations remain fair, interpretable, and consistent, allowing for the overall quality (Equation (1)) to be enhanced by improving individual IT and Health factor scores, rather than by modifying their predetermined weights. Accordingly, the higher the scores, the better the quality. Table 5 illustrates positive impacts on the overall quality of AI/ML-SaMD (Q_AI/ML-SaMD_) based on the scores, while keeping the weights unchanged.

**Figure 2:**
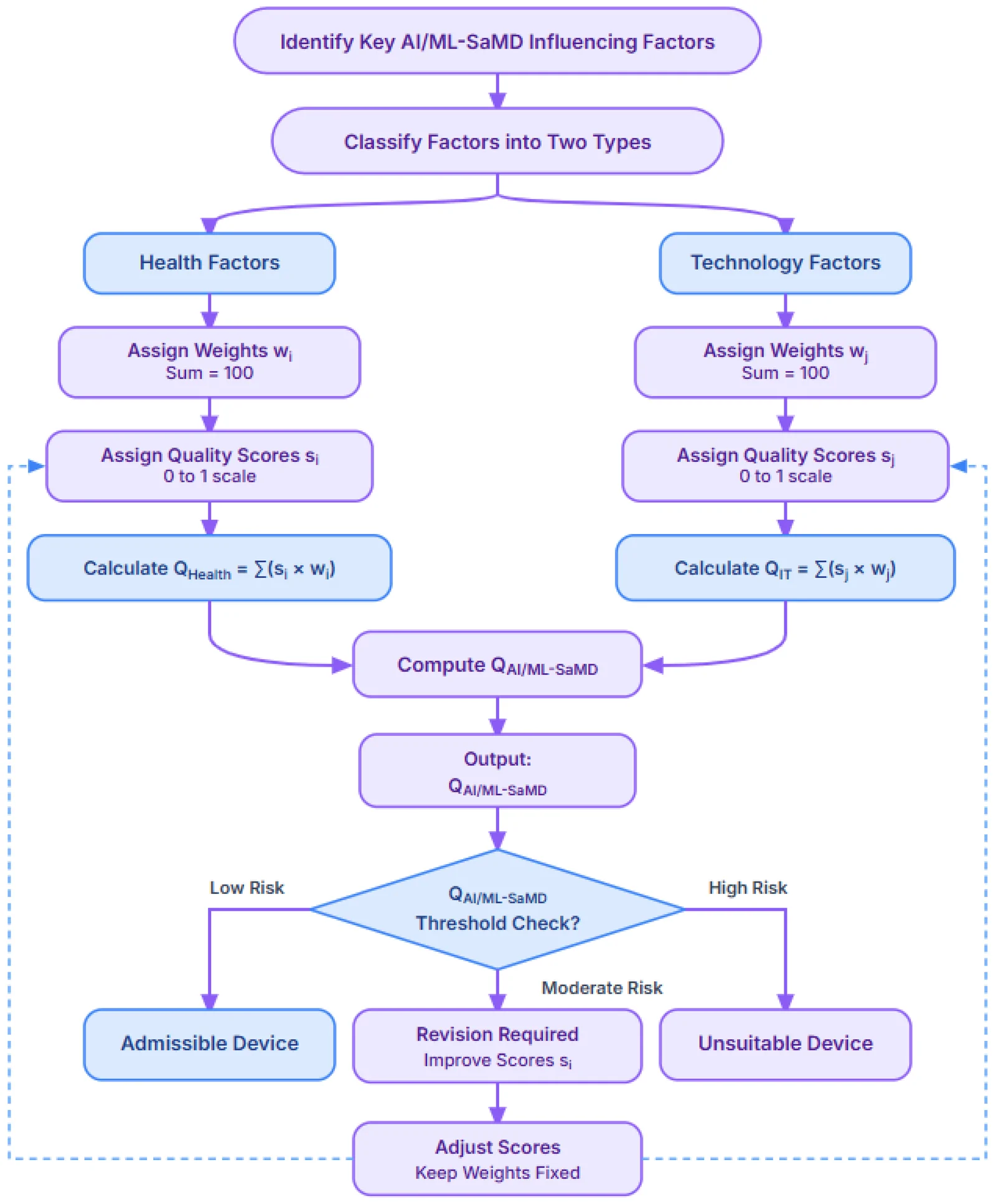
Guidance on the application of the metric in industry.

To clarify weight and score assignment in Table 4, Figure 2 illustrates a flowchart that visually captures the sequential, weighted, and averaged approach we described, and includes the iterative improvement step mentioned earlier. Section 5.4 applies the same metric to a real-world system using evidence-based scoring.

### 5.2. Sensitivity Analysis of Weight Assignments

To assess the robustness of the Q_AI/ML-SaMD_ metric against potential variations in stakeholder priorities, a sensitivity analysis was conducted. The purpose is to determine whether the qualitative classification of an AI/ML-SaMD system (e.g., “Unsuitable,” “Revision Required,” or “Admissible”) remains stable when the importance of weights w_i_ are perturbed within a plausible range.

The baseline weights from the illustrative example in Table 4 served as the reference. A one-factor-at-a-time sensitivity analysis was performed, wherein the weight of a single influential factor was increased or decreased by ±20%—a variation representing substantial divergence in expert opinion. To preserve the constraint that the sum of weights within each category (IT or Health) must equal 100, the remaining weights in the affected category were adjusted proportionally. The quality scores si were held constant at their original values from Table 4, isolating the effect of weight uncertainty. The Q_IT_, Q_Health_, and Q_AI/ML-SaMD_ values were recalculated for each scenario.

Table 6 summarizes the results. The baseline scenario yields Q_AI/ML-SaMD_ = 0.297, corresponding to an “Unsuitable” classification per Table 1.

The analysis reveals that the Q_AI/ML-SaMD_ score varies within a narrow interval of approximately 0.287 to 0.303 across all individual weight perturbations of ±20%. Importantly, the “Unsuitable” classification remains unchanged in every scenario. This consistency demonstrates that the metric’s outcome is not unduly sensitive to the specific weight values assigned in the illustrative example. Even with significant simulated disagreement over the relative importance of key factors—such as prioritizing Regulatory Approval over Algorithmic Bias, or vice versa—the low performance scores s_i_ drive a consistently low overall assessment. Consequently, the Q_AI/ML-SaMD_ metric provides a stable and robust evaluative framework suitable for supporting regulatory and development decisions, even in the presence of uncertainty or variability in stakeholder-derived weights.

Beyond weight perturbations, the sensitivity of the metric to score variations and combined uncertainty was examined. In the first extension, each score (si) was varied by ±10%, while weights remained fixed. The resulting Q_AI/ML-SaMD_ ranged from 26.5 to 31.2, with the “Unsuitable” classification preserved in all cases. In the second extension, simultaneous perturbations of ±10% were applied to both weights and scores (representing realistic uncertainty in evidence review). The minimum and maximum Q_AI/ML-SaMD_ values remained within the 0–60 “Unsuitable” range, confirming robust classification stability (shown in the last row of Table 5).

### 5.3. Expert-Based Validation

To further validate the practical relevance and face validity of the proposed Q_AI/ML-SaMD_ metric, an expert review was conducted. The metric’s framework, including its factor classification, weighting rationale, and calculation methodology, was distributed to six domain specialists. The expert panel comprised faculty members specializing in Computing (n = 4), Engineering (n = 3), and Health Informatics (n = 2), with multiple experts holding overlapping specializations. Five of the six experts (83.3%) expressed agreement with the metric’s structure and utility. The dissenting expert raised concerns regarding the interpretability of the weighting scheme and the generalizability of the illustrative example, which we have addressed by adding a detailed weight derivation explanation (Section 5.1) and an evidence-based case study (Section 5.4). The agreement level suggests strong face validity, though the small sample size is acknowledged as a limitation.

### 5.4. Empirical Application to the FDA-Authorized IDx-DR System

Unlike the illustrative example which used assumed scores, this application uses evidence-anchored scores derived from public regulatory and clinical documentation, providing a concrete test of the metric’s external validity and practicality. To address the need for real-world empirical demonstration highlighted by peer-review feedback, this section applies the proposed Q_AI/ML-SaMD_ metric to IDx-DR, an FDA-authorized autonomous AI-SaMD for diabetic retinopathy (DR) screening. IDx-DR was selected as a representative case study because it is among the earliest and most widely cited fully autonomous AI-SaMD systems, supported by a prospective multicenter clinical trial and formal regulatory authorization. The system received FDA De Novo approval in April 2018 (DEN180001) and subsequent 510(k) clearances, enabling autonomous detection of more-than-mild diabetic retinopathy (mtmDR) in primary care settings [28,29].

The ten quality factors defined in Table 3 were evaluated using publicly available regulatory documents and peer-reviewed clinical publications associated with IDx-DR. Unlike the illustrative example presented earlier, the scores assigned in this case study are evidence-anchored rather than hypothetical. Regulatory Approval status was derived directly from FDA documentation [29]. Research challenges and data sufficiency were assessed based on the scale of training data and the size and diversity of the prospective multicenter trial (900 patients) [30]. Liability and accountability were evaluated using the system’s autonomous operational designation under FDA regulation [29]. Human-centric factors were assessed through documented usability and human-factors validation testing required for regulatory clearance [29].

IT-related factors were similarly derived from published sources. Model transparency was evaluated based on the reported modular architecture involving lesion-based deep learning detectors and a decision fusion model (rather than an opaque “black-box”) [31]. Algorithmic bias was assessed through subgroup analyses across demographic groups (e.g., age, race, sex) in the pivotal clinical trial, which did not reveal performance degradation across major subgroups [30,32]. Performance and security were evaluated using the published sensitivity (87.2%) and specificity (90.7%) for mtmDR detection, imageability rate (96.1%), and FDA-required cybersecurity and system controls as described in the FDA De Novo summary [29,30]. Integration and interoperability were evaluated based on evidence that the system is designed to function with a specific fundus camera (Topcon NW400) and dedicated client software, with no broad native EHR integration reported [29,31]. Continuous learning and evolution were assessed based on the fact that the algorithm is “locked” for clinical use; any updates or retraining require new regulatory submission [29]. Software business maturity was evaluated through documentation of vendor-managed updates, licensing, and post-market surveillance obligations inherent to FDA-cleared medical devices [29,33].

According to the benchmark thresholds (Table 1), a Q_AI/ML-SaMD_ value of 0.823 places IDx-DR in the “Admissible for clinical validation (Good Quality–Low Risk)” category. This classification aligns with IDx-DR’s real-world regulatory status, clinical deployment, and documented diagnostic performance, supporting the external validity and practical relevance of the proposed metric. This case study demonstrates that the Q_AI/ML-SaMD_ metric can be operationalized using publicly available evidence, yielding a score (0.823) that correctly classifies IDx-DR as ‘Admissible.’ This alignment with the device’s real-world regulatory status provides strong empirical support for the metric’s external validity and reinforces its potential as a practical benchmarking tool for regulators and developers.

The scores (s_i_) in Table 7 were assigned using the following rubric to ensure reproducibility:

- 0.00–0.50: Significant deficiency or non-compliance (e.g., no evidence of regulatory approval), severe bias reported, major performance failure)
- 0.51–0.75: Partial compliance or moderate evidence (e.g., some documentation but not comprehensive, acceptable but suboptimal performance)
- 0.76–0.89: Strong evidence with minor gaps (e.g., robust data, clear documentation, minor limitations)
- 0.90–1.00: Exemplary evidence meeting all criteria (e.g., full regulatory approval, comprehensive validation, no reported issues)

**Table 7:** Evidence-based application to IDx-DR.

#	Factor	Evidence Basis	s_i_	w_i_	s_i_ × w_i_
A. Health (Medical) Quality Factors					
1	Regulatory Approval	FDA De Novo (2018) + 510(k) clearance for clinical use [28,29]	0.95	40	38.0
2	Research Challenges (Data)	Large-scale training and data (≥900 patient prospective trial, diverse cases) [30]	0.88	30	26.4
3	Liability & Accountability	Fully autonomous designation under FDA regulation—a defined responsibility framework defined [29]	0.82	20	16.4
4	Human-Centric Factors	Human-factors/usability validation required and conducted during approval [29]	0.88	10	8.8
**Q_Health_ = 0.896**					
**B. IT Quality Factors**					
1	AI Model/Transparency	Modular lesion-detector + decision-fusion architecture (not a pure black-box) [31]	0.72	22	15.8
2	Algorithmic Bias	Subgroup performance tested across demography; no major dropouts reported [30,32]	0.83	20	16.6
3	Performance & Security	Sensitivity 87.2%, Specificity 90.7%, imageability 96.1%; FDA cybersecurity controls described [29,30]	0.92	18	16.6
4	Integration/Interoperability	Requires specific fundus camera + client software; no public broad EHR integration [29,31]	0.62	16	9.9
5	Continuous Learning & Evolution	Algorithm locked; updates require regulatory re-submission [29]	0.65	14	9.1
6	Software Business/Maintenance	Vendor licensing, maintenance, and post-market surveillance processes in place [29,33]	0.75	10	7.5
**Q_IT_ = 0.755**					
$Q_{AI/ML-SaMD}=Q_{IT}^{\alpha }\times Q_{Health}^{\beta }=0.755^{0.5}\times 0.896^{0.5}=0.823$					

For example, Regulatory Approval (0.95) reflects FDA De Novo and 510(k) clearance with strong documentation; Integration/Interoperability (0.62) reflects the system’s functionality with a specific fundus camera but lack of broad EHR integration; Performance & Security (0.92) reflects high sensitivity/specificity [3] and FDA-described cybersecurity controls.

While IDx-DR represents a well-documented **‘good quality’** device, future research should apply the metric to devices with known quality deficiencies to assess its ability to distinguish between quality levels.

## 6. Threats to Validity and Limitations

In this section, we present the concerns that may threaten the validity of this research: internal, construct, and external validity [34].

1. **Internal validity**: the extent to which measured variables cause observable effects in the experimental data. Our paper’s core contribution is proposing and providing initial validation for a metric, not establishing a definitive cause-and-effect relationship, so internal validity is not a concern.
2. **Construct validity**: the most significant limitation concerns the construct of ‘quality’ itself. Our current model operationalizes quality through ten factors spanning IT and Health dimensions. While these factors are drawn from the most prevalent attributes in the literature [27], they are not exhaustive. Critical dimensions such as user experience (UX), detailed risk management (e.g., ISO 14971 severity/probability of harm [11]), and data governance (e.g., privacy, security protocols) are not explicitly incorporated. Consequently, the Q_AI/ML-SaMD_ score in its present form should be interpreted as a measure of technical and clinical robustness, rather than a comprehensive assessment of overall device quality. This defined scope was a necessary initial constraint, but it explicitly motivates the expansion of the model in future work.
3. **External validity**: a limitation of this study is the application of the metric to a single FDA-authorized device (Section 5.4). While this demonstrates feasibility and aligns the score with regulatory status, it does not establish generalizability. Future work will apply the metric to a diverse portfolio of at least ten AI/ML-SaMD products—including devices that have received regulatory clearance, those rejected, and those currently in development—to validate the metric’s discriminative ability across varying quality levels.

## 7. Conclusions and Future Work

The rapid adoption of AI/ML-SaMD holds immense potential to revolutionize patient care. In this paper, we proposed Q_AI/ML-SaMD_, a novel metric designed to quantify the quality of AI/ML-SaMD by synthesizing key technological and clinical factors. We validated Q_AI/ML-SaMD_ theoretically and demonstrated its practical utility through an illustrative example, a sensitivity analysis, and an evidence-based case study on an FDA-authorized AI/ML-SaMD (IDx-DR). While the study has limitations, its findings provide a foundational, quantifiable framework for assessing AI/ML-SaMD quality, paving the way for more objective and standardized evaluation in both academic and industrial settings.

As future work, our proposed Q_AI/ML-SaMD_ metric opens several promising avenues for future research, particularly in the critical areas of regulatory validation and practical application. The current formulation of Q_AI/ML-SaMD_ integrates IT and health factors. An immediate next step is to expand this model to incorporate other critical dimensions of medical device quality. Future work will focus on developing modular extensions or revised versions of the formula to systematically include factors like risk management (integrating ISO 14971 principles such as severity and probability of harm), UX and human factors (usability, workflow integration, and cognitive load), and data governance (resilience against cyber threats and compliance with data protection regulations such as Health Insurance Portability and Accountability Act (HIPAA) and General Data Protection Regulation (GDPR)). For tool development, we will investigate integrating Q_AI/ML-SaMD_ into Agile development workflows, DevOps practices, and continuous integration/continuous deployment (CI/CD) pipelines for AI/ML-SaMD development. This would allow teams to monitor the quality impact of each software commit across multiple releases, fostering a ‘quality-by-design’ approach. For empirical validation, a primary focus will be on applying the proposed metric to a diverse set of at least ten real-world AI/ML-SaMD products to demonstrate its practical utility and robustness.

## Figures and Tables

**Table 1:** Interpretation thresholds for quality assessment suggested by SFDA guidance [24]. The thresholds are presented as an illustrative example, not a fixed rule.

Q_AI/ML-SaMD_ Range	Interpretation	Comment
0–60	Unsuitable device	Low Quality (High Risk)
61–80	Revision required	Moderate Quality (Moderate Risk)
81–100	Admissible for clinical validation	Good Quality (Low Risk)

**Table 2:** Summary of how Q_AI/ML-SaMD_ relates to key frameworks, highlighting its complementary role.

Framework/Initiative	Focus	Type	How Q_AI/ML-SaMD_ Complements
FDA GMLP (FDA Good Machine Learning Practice (GMLP): https://www.fda.gov/media/153486/download, (accessed on 5 August 2026))	Process guidance for AI/ML	Qualitative, process-oriented	Provides a quantitative outcome measure to track GMLP implementation success.
ISO/IEC 25023 (https://cdn.standards.iteh.ai/samples/35747/34b91bc957f647ce8bbb2093907d7bc0/ISO-IEC-25023-2016.pdf, (accessed on 5 August 2026))	Software product quality measures	Quantitative, attribute-specific	Synthesizes multiple ISO qualities into one overall score for holistic SaMD assessment.
IMDRF SaMD (https://www.imdrf.org/sites/default/files/docs/imdrf/final/technical/imdrf-tech-140918-samd-framework-risk-categorization-141013.pdf, (accessed on 5 August 2026))	Risk categorization & principles	High-level, regulatory	Operationalizes IMDRF principles into measurable, factor-weighted scores.
Responsible AI (EU AI Act (https://artificialintelligenceact.eu/, (accessed on 5 August 2026))	Ethics, fairness, transparency	Principle-based	Quantifies ethical dimensions (bias, accountability) as part of the overall quality score.

**Table 3:** Description of the ten most significant factors (attributes/challenges) for AI/ML-SaMD quality.

# ^1^	Type	Factor	Comment
1	Health	Regulatory Approval	e.g., FDA and European Medicines Agency (EMA).
2		Research Challenges	e.g., limited access to high-quality, diverse, and sufficiently large datasets.
3		Liability and Accountability	e.g., ambiguity around who is responsible for errors.
4		Human-Centric Factors	Human/organizational factors, including stakeholder collaboration, staff training, user acceptance, and skills of data governance.
1	IT	AI Models/Transparency	Lack of transparency in “black-box” models generates outputs without interpretation, making it challenging to justify medical decisions.
2		Algorithmic Bias	e.g., demographic groups and risking unfair outcomes.
3		Performance and Security	The AI/ML-SaMD consistently delivers speedy, secure outputs across diverse circumstances.
4		Integration/Interoperability	Integrating with existing clinical systems, such as electronic health records (EHRs).
5		Continuous Learning and Evolution	New data post-deployment requires monitoring to ensure consistency with standards.
6		Software Business	e.g., updating, vendor support, documentation, licensing, and upgrading.

^1^ The terms “attribute,” “factor,” and “challenge” are used contextually to refer to the same set of quality dimensions.

**Table 4:** Calculation of the Q_AI/ML-SaMD_ value according to data in Table 3.

A. Health Quality Factors				
#	Factor	s_i_	w_i_	s_i_ × w_i_
1	Regulatory Approval	0.3	40	12.0
2	Research Challenges (Data)	0.3	30	9.0
3	Liability & Accountability	0.4	20	8.0
4	Human-Centric Factors	0.2	10	2.0
Q_Health_ = 0.310				
**B. IT Quality Factors**				
1	AI Model/Transparency	0.2	22	4.4
2	Algorithmic Bias	0.4	20	8.0
3	Performance & Security	0.3	18	5.4
4	Integration/Interoperability	0.3	16	4.8
5	Continuous Learning & Evolution	0.2	14	2.8
6	Software Business/Maintenance	0.3	10	3.0
Q_IT_ = 0.284				
$Q_{AI/ML-SaMD}=Q_{IT}^{\alpha }\times Q_{Health}^{\beta }=0.284^{0.5}\times 0.310^{0.5}=0.297$				

**Table 5:** Influence of individual scores on Q_AI/ML-SaMD_ Metric.

Scores	Q_IT_	Q_Health_	Q_AI/ML-SaMD_	Scenario
IT scores: 0.6, 0.7, 0.4, 0.9, 0.4, 0.5 Health scores: 0.7, 0.4, 0.9, 0.6	0.594	0.640	0.617	Revision required
IT scores: 0.9, 0.8, 0.95, 0.75, 0.6, 0.9 Health scores: 0.85, 0.75, 0.9, 0.9	0.823	0.835	0.829	Admissible AI/ML-SaMD

**Table 6:** Sensitivity of Q_AI/ML-SaMD_ to ±20% variations in factor weights. Weights were adjusted proportionally within their category to maintain a sum of 100.

Varied Factor (Change)	Q_IT_	Q_Health_	Q_AI/ML-SaMD_ (0–1)	Q_AI/ML-SaMD_ (0–100)	Conclusion (Per Table 1)
Baseline (Table 4)	0.284	0.310	0.297	29.7	Unsuitable
+20% Regulatory Approval	0.284	0.310	0.297	29.7	Unsuitable
–20% Regulatory Approval	0.284	0.290	0.287	28.7	Unsuitable
+20% AI Model Transparency	0.290	0.310	0.300	30.0	Unsuitable
–20% AI Model Transparency	0.278	0.310	0.294	29.4	Unsuitable
+20% Algorithmic Bias	0.296	0.310	0.303	30.3	Unsuitable
–20% Algorithmic Bias	0.272	0.310	0.291	29.1	Unsuitable
Simultaneous ±10% All Weights	0.281–0.287	0.305–0.315	0.293–0.301	29.3–30.1	Unsuitable
Simultaneous ±10% Weights & Scores	0.258–0.306	0.282–0.338	0.270–0.321	0.270–0.321	Unsuitable

## Data Availability

The tools and data used to support the findings of this study are included within the article.

## Abbreviations

The following abbreviations are used in this manuscript:

AI	Artificial Intelligence
AHP	Analytic Hierarchy Process
CI/CD	Continuous Integration/Continuous Deployment
EMA	European Medicines Agency
EHR	Electronic Health Record
EU	European Union
FDA	U.S. Food and Drug Administration
GDPR	General Data Protection Regulation
GMLP	Good Machine Learning Practice
GQM	Goal-Question-Metric
HIPAA	Health Insurance Portability and Accountability Act
IDx-DR	IDx-Diabetic Retinopathy (FDA-authorized AI system)
IMDRF	International Medical Device Regulators Forum
ISO	International Organization for Standardization
IT	Information Technology
ML	Machine Learning
mtmDR	More-Than-Mild Diabetic Retinopathy
SaMD	Software as a Medical Device
SFDA	Saudi Food and Drug Authority
SRS	Simulation for Regulation of SaMD
UX	User Experience
WGM	Weighted Geometric Mean
WHO	World Health Organization
